# Metal–Organic-Framework-Mediated Fast Self-Assembly 3D Interconnected Lignin-Based Cryogels in Deep Eutectic Solvent for Supercapacitor Applications

**DOI:** 10.3390/polym15081824

**Published:** 2023-04-08

**Authors:** Rui Lou, Qihang Cao, Taoyuan Niu, Yiyi Zhang, Yanan Zhang, Zhiwei Wang, Xiao Zhang

**Affiliations:** 1 College of Physics and Energy, Shaanxi University of Science and Technology, Xi’an 710021, China; 2 Key Laboratory of Clean Pulp & Papermaking and Pollution Control of Guangxi, Guangxi University, Nanning 530004, China; 3 Voiland School of Chemical Engineering & Bioengineering, Washington State University, Richland, WA 99354, USA

**Keywords:** lignin nanoparticles, deep eutectic solvent (DES), carbon cryogel, supercapacitor, metal–organic framework (MOF)

## Abstract

A cost-effective and sustainable method is successfully developed to produce lignin-based cryogels with a mechanically robust 3D interconnected structure. A choline chloride–lactic acid (ChCl–LA)-based deep eutectic solvent (DES) is used as a cosolvent to promote the synthesis of lignin–resorcinol–formaldehyde (LRF) gels that can self-assemble a robust string-bead-like framework. The molar ratio of LA to ChCl in DES has a significant influence on the gelation time and properties of the ensuing gels. Moreover, it is discovered that doping the metal–organic framework (MOF) during the sol–gel process can greatly accelerate the gelation of lignin. It takes a mere 4 h to complete the LRF gelation process at a DES ratio of 1:5 combined with 5% MOF. This study yields LRF carbon cryogels doped with copper that exhibit 3D interconnected bead-like carbon spheres with a prominent micropore of 1.2 nm. A specific capacitance as high as 185 F g^−1^ can be obtained for the LRF carbon electrode at a current density of 0.5 A g^−1^, and it has an excellent long-term cycling stability. This study provides a novel method of synthesizing high-lignin-content carbon cryogels with promising potential for application in the field of energy storage devices.

## 1. Introduction

Carbon aerogels (CAs), as a novel and unique porous carbon material with an interconnected structure, have abundant micropores and mesopores, a high specific surface area, controllable porosity and permeability, and higher electrical conductivity [1]. They seem to be an ideal electrode material for supercapacitors and rechargeable batteries [2]. Lignin, from woody and herbaceous biomass and as one of the most abundant plant biopolymers, has a high carbon content (up to 60%) and an aromatic structure; therefore, it can be regarded as an ideal carbon precursor [3]. Thus, lignin has long been used to synthesize thermoplastic phenolic-resin-derived CAs, which are gaining increasing interest for their excellent porous material, for supercapacitor applications [4,5].

In fact, lignin enriched with highly reactive phenolic hydroxyl groups can replace resorcinol with formaldehyde to form lignin–resorcinol–formaldehyde (LRF) gels via the sol–gel method [6]; however, industrial lignins, such as alkali lignin or enzymatic lignin, do not completely dissolve in common aqueous systems or solvents, resulting in LRF gels forming with packed and cumulated microsphere structures [7,8]. Previous work has also demonstrated that choline chloride–lactic acid (ChCl–LA)-based deep eutectic solvent (DES) can be treated as a cosolvent to prevent the shrinkage of resulting LRF cyrogels prepared via the freeze-drying process. The obtained LRF cryogels maintain good dimensional stability with a low degree of shrinkage, highly porous structure, and low thermal conductivity [7]. Therefore, during the LRF gel-forming process, ChCl–LA-based DES has shown superior properties to alkaline solvent, which is typically used as a solvent-template system and gel precursor [9,10]. The ensuing LRF-based CAs possess a high surface area with a well-developed and three-dimensional (3D) interconnected pore texture, which can be tailored by controlling the sol ingredients and adjusting the kind of cosolvent used or other condition variables [11,12]. Besides the reaction temperature and time factors, the ingredients, concentration, and rheological properties of the cosolvent significantly influence the structure and properties of the LRF gels during the emulsion polymerization process [13]. Moreover, compared to existing industrial and biorefinery lignins (e.g., kraft lignin, ligosulphonates, alkali lignin, enzymatic lignin, etc.), lignin isolated from plant biomass with ChCl–LA-based DESs (lignin nanoparticles, LNP) has a low molecular weight and abundant functional groups as well as a uniform nanoparticle size [8,14]. These unique features of LNP contribute to its outstanding ability to replace up to 100% of resorcinol in the LRF gels’ preparation [7,15].

One big challenge in developing stable LRF gels is still a long incubation time: 1–5 days or more are usually required to complete the gelation process [7,15,16]. Many studies have found that metal salts such as copper acetate or silver acetate used as a doping agent and/or a catalyst and added into the mixture prior to gelation in alkali solvent can increase the reaction rate of phenolic sols before the gelation point, thus improving the gelation kinetics [13]. However, it has been proved that copper nitrate added into an alkali solvent blocks the formation of phenolic gelation because of Cu^2+^ oxidation of resorcinol in the solution during the cure cycle [17]. Therefore, it is well known that metal salts and cosolvents have a synergic effect on the formation of LRF gels.

Metal–organic frameworks (MOFs), as a class of crystalline materials composed of metal nodes and organic ligands, have become desirable materials in the fields of catalysis, gas separation and adsorption, hydrogen storage, and drug delivery, among others, due to their large specific surface area, pore volume, and abundant Lewis/Brøsted acid/base sites [18,19,20]. HKUST–1 (Cu–BTC, BTC = benzene–1,3,5–tricarboxylate) is a typical face-centered cubic crystal involving a 3D porous organic–inorganic hybrid framework. HKUST–1 doping in the sol–gel process can efficiently assist in constructing biomass-based aerogels with a hierarchical porous structure [21]. It has also been demonstrated that HKUST–1@carbon composite aerogels exhibit significantly enhanced activities toward gas adsorption, pressure resistance, and electrochemical performances [22].

Herein, HKUST–1 was chosen as a catalyst to experimentally confirm the rapid formation of the LRF gels via combining it with a cosolvent of ChCl–LA-based DES along with a controlled mole ratio. In the LRF gels’ preparation, the incorporation of 5% MOF combined with a DES ratio of 1:5 can accelerate the stable LRF gelation process and create a new carbon aerogel with a 3D interconnected framework of high porosity. The resultant LRF carbon electrode exhibits a high specific capacitance of 185 F g^−1^ at a current density of 0.5 A g^−1^ and an excellent cycle stability in supercapacitor applications.

## 2. Materials and Methods

The LNP sample (purity 95%, Mw 800 g mol^−1^) was isolated from wheat straw by DES treatment following our previous procedures [23]. DES with a paralleled molar ratio of ChCl to LA of 1:2, 1:5, and 1:10, respectively, was used as an available cosolvent for the sol–gel process. The aforesaid DES was prepared according to our previous method [7]. As a desired catalyst, HKUST–1 ([Cu_3_(TMA)_2_(H_2_O)_3_], C_18_H_6_Cu_3_O_12_, Mw 606) was synthesized by the solvothermal method [16] and then added into the sol–gel mixture. The doping amounts of MOF were 5% and 10% based on lignin weight.

### 2.1. Synthesis of LRF Cryogels

The LRF gels were prepared by the sol–gel polymerization reaction of LNP and resorcinol (98% purity) with formaldehyde (37 wt.% in water) in DES (4 mL) as well as HKUST–1 added as a catalyst, according to the recipes in Table 1. The aforesaid mixtures were stirred to form a homogeneous suspension, then incubated at 90 °C until gelation formed. After cooling, the wet LRF gels were exchanged by a *t*–butanol/water mixture to remove remaining DES, then were frozen and dried in a freeze dryer under −60 °C and 0.2 mbar to remove solvents. Finally, the dried LRF cryogels were obtained.

### 2.2. Preparation of LRF Carbon Cryogels

The dried LRF cryogels were pyrolyzed on a tube furnace under N_2_ flow (200 mL min^−1^), heated from room temperature to 800 °C at a heating rate of 5 °C min^−1^, and kept for 2 h, to produce the resultant LRF carbon cryogels. The flow chart of the LRF carbon cryogels’ preparation is shown in Figure S1.

### 2.3. Characterizations

Morphology and composition of the samples were characterized using SEM (EM S-4800, Tokyo, Japan) coupled with energy dispersive spectroscopy (EDS). All the specimens were gold-sputtered and tested at an accelerating voltage of 15 kV.

Microstructure of the LRF carbon cryogel was characterized using TEM (Tecnai G2 F20 S-TWIN, FEI, USA).

FTIR tests were performed on a Vertex 70 spectrophotometer (Bruker, Germany) equipped with an attenuated total reflectance (ATR) cell. The wavenumber region ranged from 4000 to 400 cm^−1^ with a resolution of 4 cm^−1^. Each specimen was tested twice.

XRD patterns were recorded on a D8 Advance diffractometer (Bruker, Germany) using Cu K*α* radiation at a 2*θ* angle range of 2–40°, with a scanning step of 0.01° and a scanning rate of 2° min^−1^.

TGA was tested on a TGA–55 instrument (TA, New Castle, DE, USA). Each sample ran from 30 to 800 °C at a heating rate of 10 °C min^−1^ under N_2_ atmosphere.

N_2_ adsorption–desorption isotherms were determined using an ASAP 2460 automated gas sorption analyzer (sn:506, Atlanta, GA, USA). The specific surface area (SSA) was calculated by the Brunauer–Emmett–Teller (BET) method, the pore size distribution (PSD) by the Barret–Joyner–Halenda (BJH) model, and the micropore distribution by the t-plot method.

### 2.4. Electrochemical Measurements

Electrochemical measurements were carried out on a CHI660E electrochemical workstation (CH, Shanghai, China) at room temperature with 6 M KOH electrolyte in a typical three-electrode cell with Ag/AgCl as reference electrode and platinum wire as counter electrode. The working electrode was produced with nickel foam pasted with the homogeneous mixture of the LRF carbon cryogel with acetylene black and the binder (polytetrafluoroethylene, PTFE), with a mass ratio of 8:1:1. The cyclic voltammetry (CV) tests were performed within a potential window of −1 to 0 V at a scan rate range from 5 to 50 mV s^−1^, the galvanostatic charge–discharge (GCD) curves were obtained at current density of 0.5 to 5 A g^−1^, and the electrochemical impedance spectroscopy (EIS) worked at an open-circuit voltage in a frequency range of 10^−2^–10^5^ Hz. The specific capacitance was calculated by Equation (1) [24].

(1) $$C=\frac{I_{d}\times ∆t}{∆U\times m}$$

where *C* is the gravimetric specific capacitance (F g^−1^), *I_d_* is the discharge current (A), ∆*t* the discharge time (s), ∆*U* the potential interval (V), and *m* the mass of active material on the electrode (g).

## 3. Results

### 3.1. Effect of DES Ratio on the LRF Gelation

Given the previous success, the optimized 75% lignin-to-resorcinol replacement was used for the preparation of the LRF cryogels [7]. In this work, the LRF cryogel synthesis was assisted with a controllable ratio of DES cosolvent and doping MOF catalyst (Table 1). As an outstanding alternative cosolvent to replacing the aqueous phase, the ChCl–LA-based DES exhibited an excellent dissolving capacity in lignin during the LRF sol–gel process that accelerated the formation of the well-dispersed LRF suspensions. It is apparent that increasing the LA ratio in DES can significantly reduce the gelation rate of the LRF gels. The gelation time of the resultant LRF gels can be shortened from 48 h to 6 h by increasing the LA ratio in DES from 1:2 to 1:10. As an acid catalyst, LA could enhance the LRF polymerization and advance the gel’s point for the aggregation of primary chains as well as promote the growth of branched chains into a 3D crosslinked gel framework. Moreover, a ChCl–LA-based DES, as an alternative to water solvents, can offer good hydrogen bond reciprocity between carboxyl groups and choline N^+^ ions, providing a proper proton acid catalyst and structure-directing agent (SDA) for fast gelation of the LRF [15,25].

**Figure 1:**
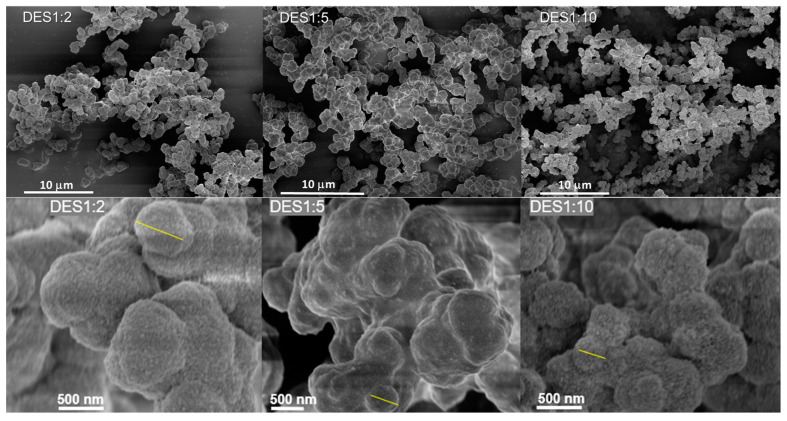
SEM images of the LRF cryogels with different DES ratios (10 µm and 500 nm).

SEM images of the LRF cryogels showed that LNP with formaldehyde could self-assemble into a highly robust 3D interconnected framework in the DES cosolvent. As the molar ratio of LA in the DES increased from 1:2 to 1:10, the individual nanosphere size in the LRF framework became smaller. Obviously, at a DES ratio of 1:5, the small nanoparticles of the LRF gel would be liable to agglomerate and form larger clusters with a self-assembled 3D interconnected framework by intermolecular van der Waals forces and hydrogen bonds in the DES system [15]. By further increasing the molar ratio of the DES to 1:10, however, a loose and ramshackle structure and framework of the LRF gel can be observed (Figure 1). Studies have revealed that the viscosity and electrical conductivity of DES depend on the ion concentration supplied by ChCl and molecular interactions, such as Coulomb attraction of cations and anions [26]. An increase in the LA to ChCl ratio would result in a decrease in the electrical conductivity and viscosity of the overall DES system [27]. Moreover, an excessive amount of LA in the DES also forced lignin depolymerization [28]. Despite an increased DES ratio of 1:10 shortened gelation time, this change would also bring a negative effect to the porosity and stability of the resulting LRF gels.

### 3.2. MOF Catalysis of LRF Cryogels Gelation

**Figure 2:**
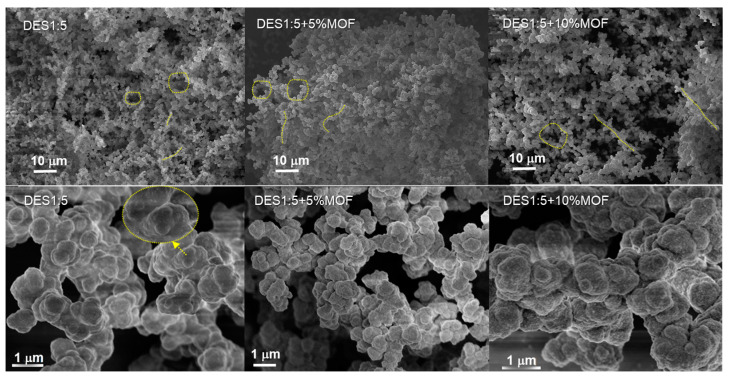
Typical SEM images of the LRF cryogels doped by MOF at DES1:5 (10 µm and 1 µm).

As mentioned earlier, HKUST–1 has been shown to improve the porosity of gel-derived carbon, which could also introduce new electro-functionality to the gels [22]. Thus, we tested the supplementation of HKUST–1 (5% and 10% MOF-based lignin mass) into the LRF cryogels. As shown in Table 1, the MOF addition significantly shortened the gelation time, even at a lower ratio of LA to ChCl, which reduced from 48 h to 16 h and 9 h, respectively. Thus, doping with HKUST–1 facilitated gelation reaction kinetics and lowered the activation energy of the polymerization reaction to produce the LRF gels [13]. Importantly, 5% of MOF load significantly shortened the gelation time three-fold at DES ratios of 1:2 and 1:5. At a higher DES ratio (i.e., 1:10), there was no obvious difference in gelation time between 5% and 10% of MOF load. It can be seen from the SEM images, despite the gelation time being short, that there was no pore structural change of collapse in the LRF cryogels after doping with MOF (Figure 2).

In the previous study, it was observed that 2,4–dihydroxy–benzoic acid (DHBA) combined with copper nitrate used in an alkali solvent during the sol–gel process effectively incorporated metal species into organic aerogels, whereas when only copper nitrate was added into a sol–gel solution it was unable to form phenolic gels [17]. More importantly, incorporating MOF during the preparation of LRF carbon cryogels has not been attempted in the past. The representative MOF of HKUST–1 can be synthesized by copper(II) cations (Cu^2+^) and BTC anions to form a neutral net structure where the paddle-wheel secondary building unit has four carboxylate groups in four BTC ligands that link together with two copper ions [26]. It is proved that HKUST–1 dissolved readily in the DES cosolvent at room temperature (Figure S2), then the initial blue color of HKUST–1 turned bright green in a homogeneous solvent phase, attributed to each copper ion with an unsaturated binding site in MOF dissociating into Cu^2+^ and to a chelating reaction with chloride ions in the DES system. Benefitting from the introduction of Cu^2+^ from HKUST–1 hydration, the presence of methoxy, phenolic hydroxyl, and carboxyl groups in LNP structure allowed Cu^2+^ to be coordinated, thus Cu^2+^ acted as metal cation catalyst in the sol–gel process to facilitate the condensation and polymerization of lignin with formaldehyde [20]. Therefore, copper species can be effectively incorporated into the LRF cryogels in the sol–gel polymerization process. Herein, this work demonstrated, for the first time, the promising potential of applying MOF as a mediated catalyst to improve the formation of LRF cryogels.

**Scheme 1:**
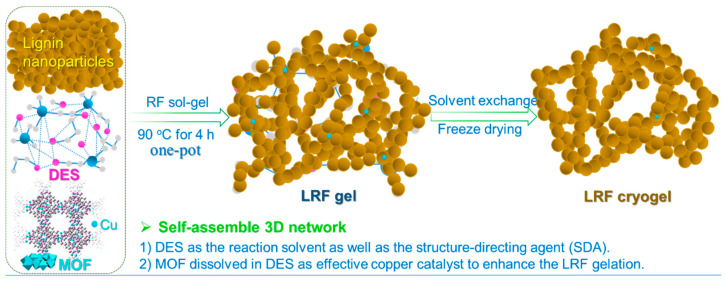
Schematic preparation of LRF cryogel formation in DES cosolvent mediated by MOF.

Under the desired DES ratio of 1:5, the control tests with MOF doped as a catalyst demonstrated that the nanosphere size of primary particles in the LRF gels’ framework gradually decreased with an increase in MOF load. Moreover, the nanoparticle size and branch chains of the LRF gels’ interconnected framework depended on the copper content in the matrix. With an amount of MOF of 10%, the produced branch chains were longer and nanoparticle clusters became larger (Figure 2). This suggested that the addition of MOF could enhance the porous structure and property of the LRF cryogels as well as the resultant carbonaceous microstructure, such as the surface areas, pore volumes, and electrochemical performances [17,23]. Most notably, there are no visible MOF crystals or precipitated copper particles on the surface of the LRF cryogels, revealing that the copper ions uniformly dispersed in the gel particles probably bound to carboxylate moieties. Scheme 1 illustrates the proposed schematic diagram of the LRF cryogel formation in the DES cosolvent mediated by MOF. During the LRF sol–gel process, lignin and HKUST–1 completely dissolved in the DES medium successively with formaldehyde to produce sols as a Newtonian fluid, then further formed the aggregates of the crosslinked gels under the low temperature of 90 °C. The spherical, dispersed lignin nanoparticles are crosslinked mainly by intermolecular van der Waals forces and hydrogen bonds in the DES to self-assemble a robust and defined structure with a 3D interconnected string-bead-like framework.

**Figure 3:**
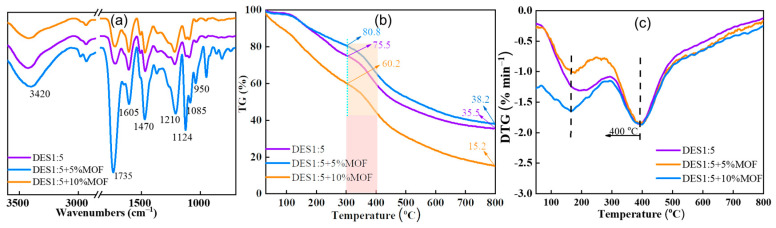
FTIR spectra (**a**) and TG and DTG curves (**b**,**c**) of the LRF cryogels at DES1:5.

The polymerization reactions in the gelation process almost occurred with the C_5_ substitution of lignin aromatic nucleus from guaiacyl (G) and *p*–hydroxylphenyl (H) subunits or with the lignin propenyl side chains [7,29]. The functional groups presented in three LRF cryogels were tracked using FTIR (Figure 3a), apparently the sample of DES1:5+5%MOF had the strongest absorption peaks. The region of 1850 to 1000 cm^−1^ can provide the pivotal structural information on the C–O stretch bond on the side chain and for C=C on aromatic rings. The peak around 1735 cm^−1^ in LNP, attributed to C=O stretching of unconjugated ketones and carbonyl groups, was more visible in the spectra of the LRF gels. This observation suggests that aldehydes, which initially presented in lignin, might have contributed to its crosslinking and/or reacted with resorcinol in the gelation reaction to a large extent [15]. For LNP, the characteristic absorption peak of the aromatic ring in the lignin structure at 1512 cm^−1^ disappeared (Figure S3), but the absorbance at 1605 cm^−1^ assigned to C=C stretching was still observable after the gelation. Furthermore, the apparent structural changes in the LRF cryogels occurred in the aromatic C=C stretch region at 1470 cm^−1^. Another characteristic of lignin-based gels was also supported by the peak of the secondary alcoholic groups at 1085 cm^−1^ and the strong intensity of the band at 950 cm^−1^ that was ascribed to the C–O stretch of resorcinol [30]. In contrast, it is noted that the characteristic peaks of HKUST–1 at 1640, 1370, 1105, and 725 cm^−1^ disappeared in the FTIR spectra of the crosslinked LRF cryogels, thereby confirming that the copper MOF had disassembled in the DES as an ion–hydrogen–bond–donor supramolecular complex.

The thermal stability of the synthesized LRF cryogels was determined. As illustrated in Figure 3b,c, it is clearly displayed that the LRF sample of DES1:5+5%MOF had an improved thermostability that was close to that of LNP (Figure S4), proving that the incorporation of HKUST–1 with 5% load indeed strengthened the structural network of the ensuing cryogel, and its yield of the residual solid remained above 80% after 300 °C. However, HKUST–1 with 10% load in the LRF gels showed a much faster degradation rate before 300 °C, and especially above 400 °C. As a whole, these LRF cryogels prepared at DES1:5 had a significantly lower thermostability compared to LNP itself. It appeared that the presence of oxygen-containing functional groups (e.g., hydroxyl, ketone) in the crosslinked LRF cryogels could cause reduced thermal stability [7,15]. The N_2_ adsorption–desorption isotherms on the LRF cryogel of DES1:5+5%MOF showed a type Ⅲ H_4_ hysteresis loop (Figure S5), suggesting that the LRF cryogel had a slit-like pore structure formed by particle aggregation with major macropores and mesopores. Moreover, the multi-layer adsorption occurred at a low relative pressure, followed by capillary condensation as the pressure increased (P/P_0_ > 0.8). The SSA was 33.78 m^2^ g^−1^ for the BET model, much higher than that of other LRF cryogels (3.38 m^2^ g^−1^ for DES1:5, 4.67 m^2^ g^−1^ for DES1:5+10%MOF).

### 3.3. Characterization of LRF Carbon Cryogels

**Figure 4:**
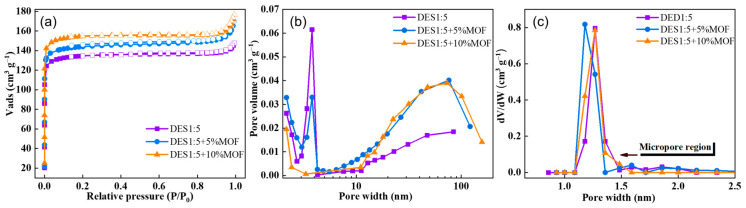
N_2_ adsorption–desorption isotherms (**a**) and PSDs (**b**,**c**) of the LRF carbon cryogels.

The resultant LRF carbon cryogels were obtained at 800 °C through slow pyrolysis. The highest solid yield of 38.8% was obtained from the LRF cryogel of DES1:5+5%MOF, which represented the highest stability framework structure among these LRF cryogels. In contrast to the initial LRF cryogel, the SSA and porosity of the ensuing LRF carbon cryogels were significantly improved. Figure 4a plots the N_2_ adsorption–desorption isotherms of the LRF carbon cryogels at 196 °C. They belonged to type I of the IUPAC classification [31], typical of microporous carbon solids, resulting from the multi-layer adsorption of several nanometer micropores, rather than mono-layer at a low relative pressure (P/P_0_ < 0.1). The N_2_ uptake obviously increased at a higher relative pressure (P/P_0_ > 0.9) after micropore filling. In the condensation process, the curved surface changes with the increase in P/P_0_ in capillary pores was reversible; there was a change in the curved surface formed by the evaporation process when P/P_0_ decreased, resulting in the adsorption/desorption isotherms overlapping without a hysteresis circle [13]. Figure 4b demonstrates that a micropore (1–2 nm) and mesopore (10 nm) dominated the pore distribution with a fraction of the macropore (70 nm), thus the LRF carbon cryogels showed a typical hierarchical porous nanostructure.

It has been reported that the carbonous material with a hierarchical porous structure was an ideal electrode for improving the supercapacitor performance, suggesting the that the micropores (<2 nm) can accommodate charge and strengthen capacitance, the mesopores (2–50 nm) favored ion transport, and the macropores (>50 nm) help to allow ion buffering [32]. Furthermore, the micropores distributed in a 1–2 nm width played a valuable role in adsorption volume, and around 83% surface area and over 80% pore volume could be offered by the micropores (Figure 4c). The abundant micropores generated in the ensuing LRF carbon cryogels in a high-temperature pyrolysis process contributed to an excellent high-rate capacitance. Moreover, the SSA of the LRF carbon cryogels increased from 421 to 482 m^2^ g^−1^ by BET theory, along with the MOF load increasing in the LRF cryogel precursors (Table 2). For example, the LRF carbon cryogels with 5% MOF gained a large pore volume (0.26 cm^3^ g^−1^) as well as a micropore area of 408 m^2^ g^−1^ and a micropore volume of 0.21 cm^3^ g^−1^ calculated by the t–Plot method, which suggests that HKUST–1, as a superb catalyst, had a significant influence on the microstructure of the LRF carbon cryogels, consistent with the results of doping metal salts (Fe, Mn, Cu, etc.) into phenolic mixtures, which can improve the porous structure of carbon aerogels [29,33]. It can be seen from Table 2 that the LRF carbon cryogel of DES1:5+5%MOF exhibited the highest mesoporous rate and the lowest micropore ratio. Therefore, the LRF carbon cryogels belonged to a low-density, nanoporous, non–crystalline solid whose microstructure can be controlled via the variables of reaction conditions.

**Figure 5:**
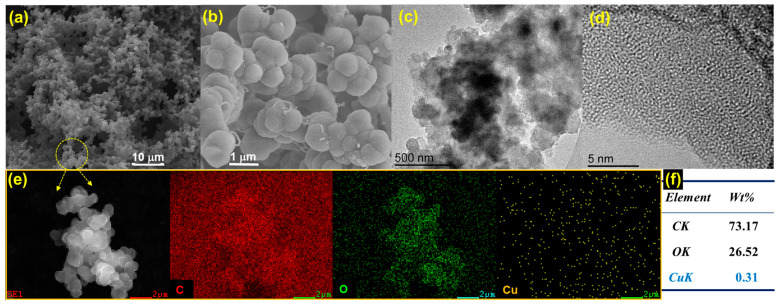
Microscopic structures and compositions of the LRF carbon cryogel (DES1:5+5%MOF): (**a**,**b**) SEM images; (**c**,**d**) TEM and HRTEM images; (**e**,**f**) elemental content by EDS mapping.

With the help of SEM–EDS and XRD, the compositions, structures, and properties of carbon cryogels were analyzed. SEM images (Figure 5a,b) showed that the LRF carbon cryogel had a 3D interconnected framework in self-assembled bead–like chains crosslinked with numerous carbon nanospheres. After pyrolysis at a high temperature of 800 °C, the depolymerization of the foregoing LRF cryogels resulted in the firmly round carbon nanospheres forming stronger cohesion in the carbon chains. Overall, the obtained LRF carbon cryogel of DES1:5+5%MOF exhibited a more robust structure, interlinked with carbon nanospheres, revealing that the branched chains and 3D interconnected substrate precursor did not collapse during the thermal degradation. Moreover, the diameter of the LRF carbon nanoparticles was distributed in a narrow range of 100–500 nm, owing to shrinkage in the thermal treatment stage. In addition, the single carbon nanosphere was approximately 50 nm in diameter, which gathered the cluster chains to self-assemble a 3D interconnected bead-like structure. TEM and HRTEM images (Figure 5c,d) further confirmed that the LRF carbon cryogel had a stable 3D interconnected network with a macroporous and microporous structure from self-assembled nanospheres. The observed LRF carbon nanospheres had representative worm-like micropores with an amorphous structure of short-range order and some disorder features. Furthermore, the XRD pattern demonstrated that the LRF carbon cryogel consisted of crystallographic planes of (002) and (100), corresponding to the respective 2*θ* position of 26.5 ° and 43.2 ° (Figure S6) [34]. However, the signal of the copper was not detected due to a trace of metallic copper in the LRF carbon cryogel [17]. Element mapping (Figure 5e,f) showed that the LRF carbon cryogel mainly contained carbon (73.17%) and oxygen (26.52%), as well as trace amounts of copper (0.31%), indicating that the copper in the MOF successfully hybridizes within the LRF carbon. These existing oxygen groups could also improve carbon wettability, together with the copper, contributing to capacitance by Faradic reactions as well as pseudocapacitance [1,6].

### 3.4. Electrochemical Performances of LRF Carbon Cryogels

**Figure 6:**
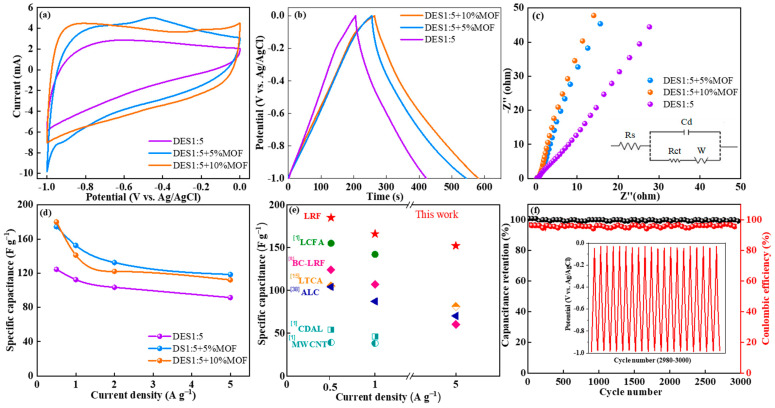
Electrochemical performances of the LRF carbon electrodes in 6 M KOH electrolyte: (**a**,**b**) representative CV and GCD curves; (**c**) Nyquist curves and the inset of equivalent circuit diagram; (**d**,**e**) mass-specific capacitances; (**f**) cycling stability for 3000 cycles at 5 A g^−1^).

On the basis of the foregoing, the selected LRF carbon cryogels at a DES ratio of 1:5 was determined to the energy storage capacity, and the electrochemical performances were tested by CV, GCD, and EIS in a three-electrode system. CV curves for the LRF carbon electrodes were measured with a potential window range from −1 to 0 V at a series of scan rates of 5–50 mV s^−1^ (Figure 6a). Obviously, at a scan rate of 50 mV s^−1^, all curves displayed a quasi-rectangular shape with weak reversible redox peaks, especially the LRF carbon cryogels doped with MOF, which showed a bigger integrated area of CV curves, meaning they had an excellent rate capability and a superior charge response speed for electrical double-layer capacitance (EDLC) as well as fast electron transport for pseudocapacitance behaviors, which can be attributed to the doped copper and oxygen in carbon cryogels. In addition, the GCD curves of all samples at 0.5 A g^−1^ showed a quasi-symmetric isosceles triangle (Figure 4b), illustrating the typical capacitance characteristics of EDLC, which demonstrated that the electrodes of the LRF carbon cryogels doped with MOF had an excellent electrochemical reversibility [35], attributed to the dedicative micropores with 1–2 nm width and the self-assembled 3D interconnected opened framework structure.

The Nyquist plot of the frequency range displayed a semicircle in the high-frequency range and a straight line in the low-frequency range as shown in Figure 6c. The high slope of the curves at low frequencies indicated that all LRF carbon cryogels possessed a small ion diffusion resistance and a high electrode conductivity and electron transfer rate. The equivalent circuit can be joined with a series of solution resistance (R_s_), charge transfer resistance (R_ct_), Warburg impedance (W), and double-layer capacitance (C_d_) [36]. In the high-frequency region, the lowest R_s_ = 0.5 Ω for the LRF carbon cryogel of DES1:5+5%MOF suggested that this electrode had the highest charge transfer efficiency and highest conductivity on the electrode–electrolyte interface. Moreover, all R_ct_ of the high-frequency curves with a faint semicircle revealed that the ion transfer in the electrolyte offered a small resistance and fast ion transport in the electrode, owing to the crosslinked hierarchical porous structure as well as a proper graphitization [37]. Electrode conductivity and ion diffusion are of great importance to the capacitance and capacitive deionization performance of electrode materials [38].

The specific capacitances of three LRF carbon cryogels were calculated based on the GCD curves (Figure S7). The introduction of MOF incorporation into the LRF carbon cryogels significantly enhanced the specific capacitance, i.e., at a current density of 0.5 A g^−1^ both LRF carbon cryogels of DES1:5+5%MOF and DES1:5+10%MOF achieved 185 F g^−1^ and 180 F g^−1^, respectively, while the DES1:5 sample without MOF gained the lowest specific capacitance of 135 F g^−1^. As the current density increased to 5 A g^−1^, the DES1:5+5%MOF sample could still maintain 142 F g^−1^, revealing that it had the highest capacitance retention rate and stability. Compared to other supercapacitor electrodes of carbon materials prepared by electrospinning, chemical activation, etc., the LRF carbon cryogels exhibited the highest specific capacitance (Figure 6e). Lignin-based carbon fiber aerogels (LCFA) prepared with the DES-based ChCl–LA by electrospinning obtained a specific capacitance of 155 F g^−1^ at a current density of 0.5 A g^−1^, much higher than that of carbonized dealkaline lignin (CDAL, 54 F g^−1^) and commercial multi-wall carbon nanotubes (MWCNT, 39 F g^−1^) [28]. Compared with the electrospinning and chemical activation methods, the preparation of LRF carbon cryogels by the sol–gel method was efficient, simple, and easy to operate. It most notable that the LRF carbon cryogels as carbon electrodes could have a much higher specific capacitance compared to LCFA, yolk–shell-structured carbon nanospheres (YSCN, 159 F g^−1^), BC–LRF carbon aerogels, lignin/TOCNF–derived carbon aerogels (LTCA), and alkali lignin carbon (ALC) [39,40,41].

Furthermore, the cycling stability of the DES1:5+5%MOF sample was tested at 5 A g^−1^ (Figure 6f), and displayed a cycling performance where the capacitance retention rate was 100% and the Coulombic efficiency was up to 97% over 3000 cycles, which demonstrated that this LRF carbon cryogel had an excellent operational stability, high reversibility, and outstanding cycling durability. This was a result of the LRF carbon cryogel having a robust 3D interconnected opened framework as well as a hierarchical pore structure, offering unimpeded ion diffusion channels [42]. Therefore, this a novel method for the easy and fast production of LRF carbon cryogel precursors through LRF self-assembly in green DES assisted by MOF. The resultant carbon cryogels, involving the advantages of a 3D interconnected hierarchical porous structure, mechanical and chemical stability, and excellent electrochemical performance, are able to be employed as electrode materials for advanced supercapacitors.

## 4. Conclusions

The proposed simple and fast method can produce LRF carbon cryogels with a 3D and highly interconnected hierarchical porous framework. The molar ratio of the DES-based ChCl–LA as a cosolvent, combined with MOF catalysis, has a synergistic effect on significantly accelerating the LRF gelation process. For the first time, the results demonstrate that the incorporation of HKUST–1 (a typical MOF) can catalyze the gelation kinetics of LRF formation and further improve the hierarchical pores’ microstructure. Therefore, by combining a 5% MOF load with a DES ratio of 1:5 can significantly prompt the formation of LRF gels as well as greatly shorten the gelation time to as fast as 4 h. The subsequent LRF carbon cryogels have shown a promising potential for application in high-performance electrochemical capacitive energy storage, exhibiting excellent performance with the specific capacitance of 185 F g^−1^ at 0.5 A g^−1^. This study provides a new approach for high-value agricultural biomass utilization and the self-assembly fabrication of biopolymer materials for energy storage.

## Figures and Tables

**Table 1:** Gelation time of the LRF gels under different conditions.

Entry#	LNP (mg)	Resorcinol (mg)	Formaldehyde (mg)	HKUST–1 (mg)	ChCl to LA	Gelation Time (h)
DES1:2	150	50	200	–	1:2	48
DES1:2+5%MOF	150	50	200	7.5	1:2	16
DES1:2+10%MOF	150	50	200	15	1:2	9
DES1:5	150	50	200	–	1:5	11
DES1:5+5%MOF	150	50	200	7.5	1:5	4
DES1:5+10%MOF	150	50	200	15	1:5	5
DES1:10	150	50	200	–	1:10	6
DES1:10+5%MOF	150	50	200	7.5	1:10	5
DES1:10+10%MOF	150	50	200	15	1:10	5

**Table 2:** The SSA and porosity of the LRF carbon cryogels at DES ratio of 1:5.

Sample	S_BET_ (m^2^ g^−1^)	V_total_ (cm^3^ g^−1^)	V_micro_ (cm^3^ g^−1^)	V_meso_ (cm^3^ g^−1^)	Pore Size (nm)
DES1:5	421	0.23	0.19	0.02	1.96
DES1:5+5%MOF	457	0.26	0.21	0.05	1.96
DES1:5+10%MOF	482	0.27	0.22	0.04	1.98

## Data Availability

The data presented in this study are available in the Supplementary Materials.

## Supplementary Materials

The following supporting information can be downloaded at https://www.mdpi.com/article/10.3390/polym15081824/s1, Figure S1: Flow chart of the LRF carbon cryogel preparation; Figure S2: A typical MOF of HKUST–1; Figure S3: FTIR spectra of the LRF cryogel (DES1:5+5%MOF), LNP, MOF and resorcinol (R); Figure S4: Thermal stability of the LRF cryogels and LNP; Figure S5: N_2_ adsorption–desorption isotherms of the LRF cryogel (DES1:5+5%MOF); Figure S6: XRD pattern of the LRF carbon cryogel (DES1:5+5%MOF); Figure S7: CV and GCD curves of the LRF carbon cryogels (DES1:5, DES1:5+5%MOF, and DES1:5+10%MOF).
