# Supplementary material for: Metal–Organic-Framework-Mediated Fast Self-Assembly 3D Interconnected Lignin-Based Cryogels in Deep Eutectic Solvent for Supercapacitor Applications
Source: Polymers (Basel). 2023 Apr 8;15(8):1824. doi: 10.3390/polym15081824 (PMC10141371; doi:10.3390/polym15081824)
Supplement: Supplementary file 1 [file polymers-15-01824-s001.zip › polymers-2272140-supplementary.pdf]

Supporting Information

Metal–Organic-Framework-Mediated Fast Self-Assembly 3D Interconnected Lignin-Based Cryogels in Deep Eutectic Solvent for Supercapacitor Applications

Rui Lou [1,*], Qihang Cao [1], Taoyuan Niu [1], Yiyi Zhang [1], Yanan Zhang [1], Guodong Wei [1], Zhiwei Wang [2] and Xiao Zhang [3,*]

1 College of Physics and Energy, Materials Institute of Atomic and Molecular Science, Shaanxi University of Science and Technology, Xi'an 710021, China
2 Key Laboratory of Clean Pulp & Papermaking and Pollution Control of Guangxi, Guangxi University, Nanning 530004, China
3 Voiland School of Chemical Engineering & Bioengineering, Washington State University, Richland, WA 99354, USA

* Correspondence: lourui@sust.edu.cn; x.zhang@wsu.edu

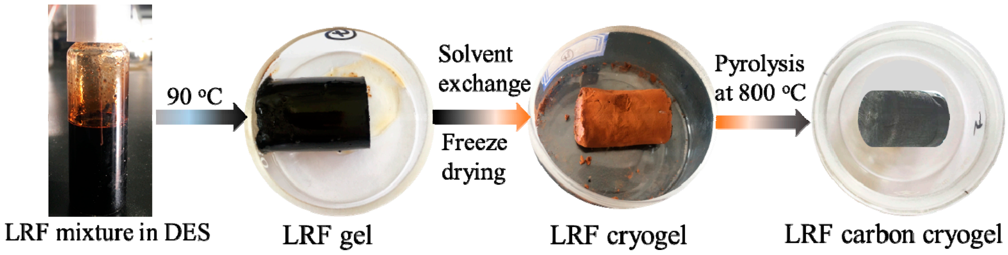

Figure S1 Flow chart of the LRF carbon cryogels preparation.

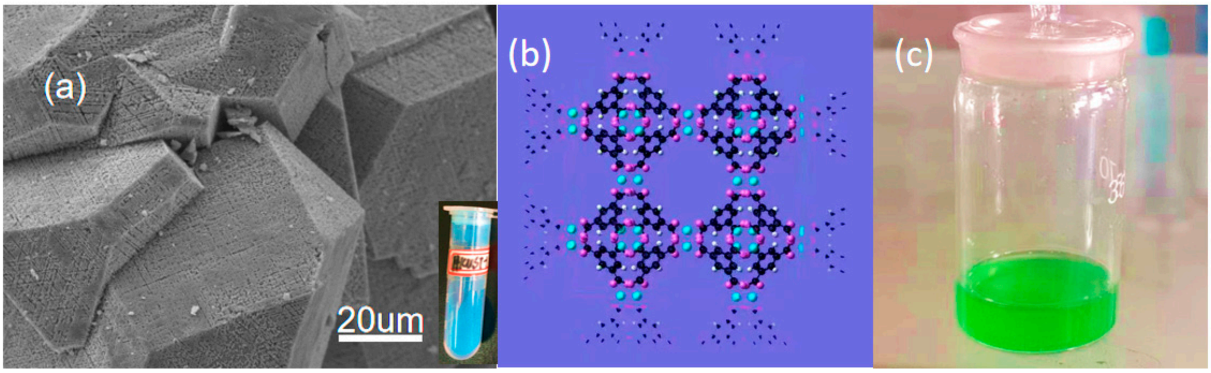

Figure S2 A typical MOF of HKUST–1. ((**a**) SEM image; (**b**) molecular lattice structure; (**c**) HKUST–1 dissolved in DES ratio of 1:5 at room temperature).

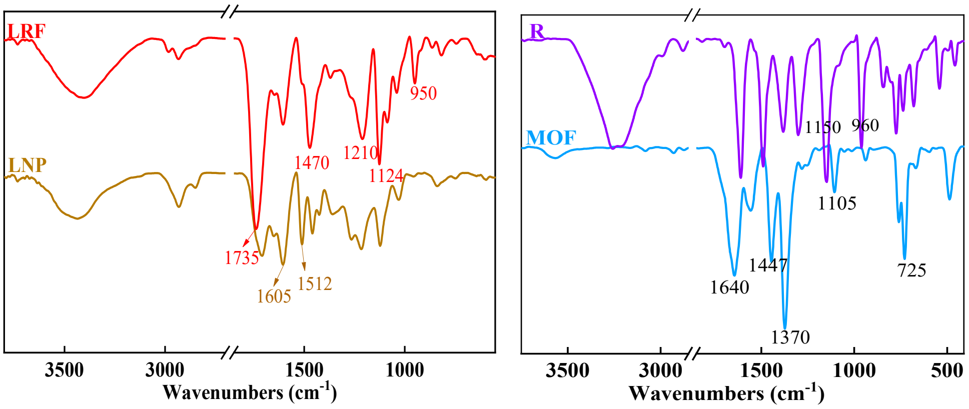

Figure S3 FTIR spectra of the LRF cryogel (DES1:5+5%MOF), LNP, MOF and resorcinol (R).

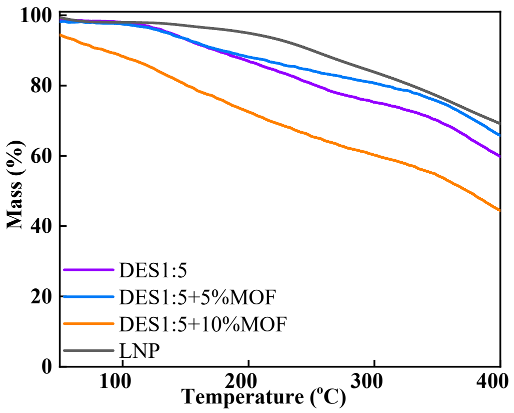

Figure S4 Thermal stability of the LRF cryogels and LNP.

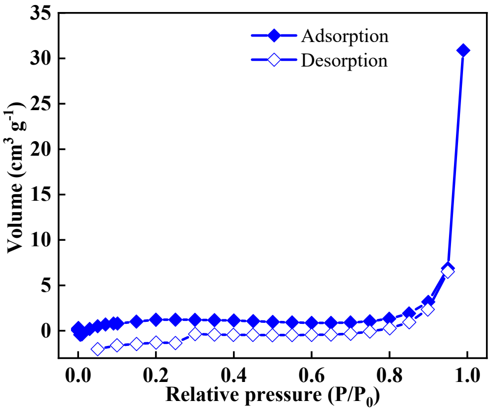

Figure S5 N_2 adsorption–desorption isotherms of the LRF cryogel (DES1:5+5%MOF).

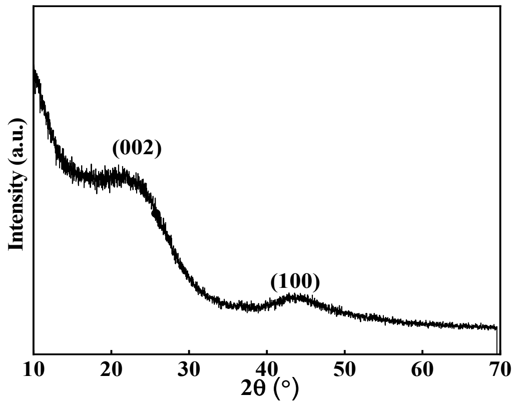

Figure S6 XRD pattern of the LRF carbon cryogel (DES1:5+5%MOF).

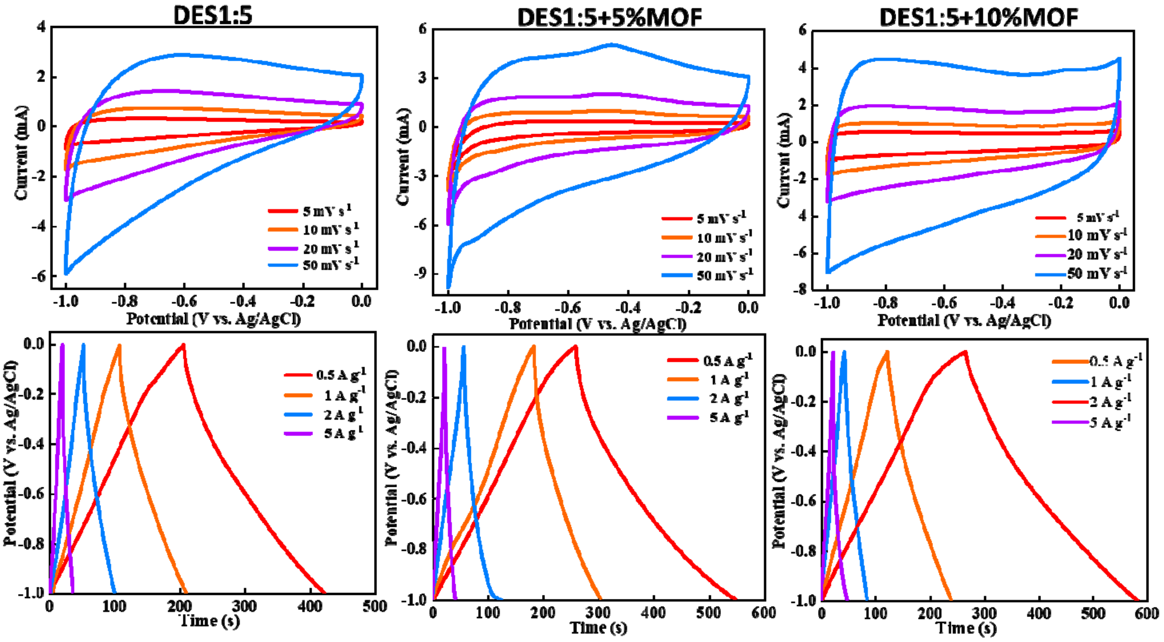

Figure S7 CV and GCD curves of the LRF carbon cryogels (DES1:5, DES1:5+5%MOF, and DES1:5+10%MOF).
